# Software Trusted Platform Module (SWTPM) Resource Sharing Scheme for Embedded Systems

**DOI:** 10.3390/s25123828

**Published:** 2025-06-19

**Authors:** Da-Chuan Chen, Guan-Ruei Chen, Yu-Ping Liao

**Affiliations:** Department of Electrical Engineering, Chung Yuan Christian University, Taoyuan 320314, Taiwan; dachuan516@gmail.com (D.-C.C.); poil123456456@gmail.com (G.-R.C.)

**Keywords:** embedded system network, measured boot, remote attestation, SWTPM, secure boot, system integrity, dTPM

## Abstract

Embedded system networks are widely deployed across various domains and often perform mission-critical tasks, making it essential for all nodes within the system to be trustworthy. Traditionally, each node is equipped with a discrete Trusted Platform Module (dTPM) to ensure network-wide trustworthiness. However, this study proposes a cost-effective system architecture that deploys software-based TPMs (SWTPMs) on the majority of nodes, while reserving dTPMs for a few central nodes to maintain overall system integrity. The proposed architecture employs IBMACS for system integrity reporting. In addition, a database-based anomaly detection (AD) agent is developed to identify and isolate untrusted nodes. A traffic anomaly detection agent is also introduced to monitor communication between servers and clients, ensuring that traffic patterns remain normal. Finally, a custom measurement kernel is implemented, along with an activation agent, to enforce a measured boot process for custom applications during startup. This architecture is designed to safeguard mission-critical embedded systems from malicious threats while reducing deployment costs.

## 1. Introduction

Embedded systems provide an attractive combination of a compact size, moderate cost, and sufficient computational power, making them suitable for a wide range of applications. These systems are frequently deployed in distributed networks, where safeguarding each unit from physical tampering and cybersecurity threats poses significant challenges. In mission-critical contexts, compromised embedded devices can cause severe consequences. Moreover, these nodes’ network resources can be exploited to form botnets or launch attacks. Due to the large scale of deployment and strict cost constraints, it is impractical to equip every unit with expensive security components. To address these issues, this paper proposes a Trusted Platform Module (TPM) sharing scheme aimed at enhancing protection against tampering and malicious use while minimizing additional deployment costs.

Ensuring the system integrity of individual nodes can be aided by TPMs [1] and cryptographic smart cards with similar functionalities [2], standardized by the Trusted Computing Group (TCG) [3]. Meanwhile, various studies have proposed intrusion detection techniques through network monitoring [4,5,6], and others have explored trace-based methods to detect Distributed Denial of Service (DDoS) attacks [7,8,9]. Motivated by these findings, this study focuses on combining TPM-enabled integrity measurement with network monitoring to protect embedded system networks.

**Figure 1:**
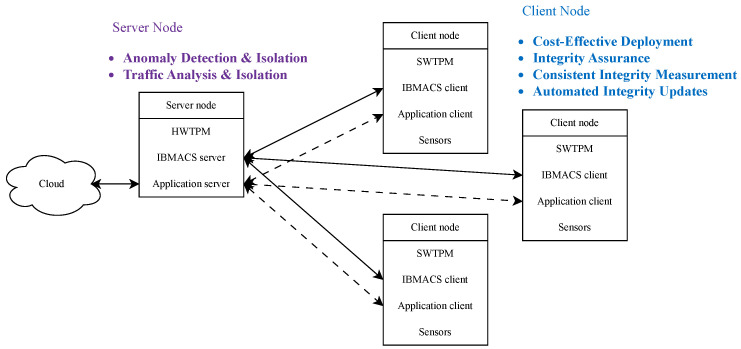
Embedded system network deployment scenario.

As illustrated in Figure 1, this study’s architecture aims to identify maliciously modified client nodes and isolate them from the network. Leveraging the IBM (Armonk, NY, United States) Attestation Client Server (IBMACS), the system realizes remote integrity reporting. Because IBMACS itself does not perform remediation, a custom agent is developed to take appropriate action upon detection of integrity violations. By default, IBMACS monitors only OS-level integrity; therefore, this study introduces a custom integrity measurement kernel to extend coverage to mission-critical applications. Additionally, a network monitoring agent is implemented to detect suspicious client node activities.

The embedded system network consists of a server node overseeing multiple client nodes. The server node acts as the central hub for network security and application data exchange, is equipped with a hardware TPM (HWTPM), and operates in a trusted environment hardened against tampering. It is also the sole node with internet connectivity, enabling selective cloud exposure. In contrast, client nodes focus on application-specific tasks such as data collection via sensors, processing, and communication with the server.

This research prioritizes the identification and isolation of compromised client nodes deployed in untrusted environments to limit their impact on the local network. Client nodes are designed with minimal physical influence, ensuring any potential abuse is contained even if network communication is severed. The framework relies heavily on TPM-enabled measured boot to verify system integrity. However, due to TPM’s limited computational resources, full system-wide modification prevention is beyond this study’s scope. Self-termination mechanisms are also excluded to avoid unstable system behavior. Crucially, the server must verify client integrity reports before permitting any interaction.

The proposed framework is grounded in the use of measured boot, a TPM-based mechanism for verifying software integrity during system startup. The system is specifically designed to defend against network-based intrusions that aim to modify onboard software, preventing compromised nodes from behaving as originally intended. However, the limited processing capacity of TPM makes it impractical to enforce complete system-wide integrity or prevent all possible modifications. Additionally, self-termination mechanisms are not included, as they could lead to erratic behavior if triggered under manipulated conditions.

The remainder of this paper is structured as follows: Section 2 reviews related works on hardware-based security, data encryption, intrusion detection, and network anomaly identification such as DDoS attacks. Section 3 introduces the foundational technologies. Section 4 details the proposed integrity maintenance system. Section 5 evaluates the approach experimentally. Finally, Section 6 summarizes conclusions and future research directions.

## 2. Related Work

Researchers and industry professionals have proposed various mechanisms to establish trust in computing environments, with a strong focus on hardware-based roots of trust and privacy-preserving authentication. Trusted Platform Module (TPM), Secure Elements (SE), Intel TXT, Trusted Execution Environments (TEEs), and Encrypted Execution Environments (E3s) represent the backbone of many such approaches. A comparative study by [10] analyzes these technologies, highlighting their respective security guarantees and trade-offs.

In the realm of attestation, the Direct Anonymous Attestation (DAA) protocol has received notable attention. Bei and Guangyuan [11] enhanced the original DAA with a more efficient anonymous attestation method leveraging a one-way function and the DDH assumption. Parno et al. [12] provided foundational insights into DAA within TPM-backed systems, while Sedighi et al. [13] extended this with support for TPM 2.0 to improve verifier-side privacy.

Privacy-preserving identity schemes using X.509 certificates and Privacy CAs have been explored for cloud-based environments [14]. These approaches focus on preventing MITM attacks while authenticating user identities. Further, research by Wang and Yan [15], and Lu et al. [16] explored trusted boot processes and authentication protocols in embedded systems lacking hardware TPMs, proposing node verification mechanisms using TPM-equipped monitors.

Beyond attestation, integrity measurement and intrusion detection have been integrated with trusted computing. Sailer et al. [1] implemented a TPM-based integrity system for Linux. Meng et al. [17] reviewed IDS designs in conjunction with blockchain, while Mantas et al. [2] employed TPM-based iterative hashing to ensure runtime integrity. Blockchain has also been utilized by Cao et al. [18] to seal health data against tampering.

On the networking front, Zargar et al. [8] categorized protocol-abusing DDoS attacks and identified points of defense. Papadopoulos et al. [7] introduced the Cossack architecture to analyze frequency patterns in traffic anomalies. Estan and Varghese [9] emphasized stream data analysis for early attack detection, and Agrawal and Tapaswi [19] tailored defensive strategies for cloud service providers.

Table 1 provides a detailed comparison of these works in terms of context, approach, and technology. For example, Cao et al. [18] focus on securing Electronic Health Records via blockchain, while Wang and Yan [15] and Lu et al. [16] propose protocol-level trust enforcement for resource-constrained embedded devices. This study aims to integrate these diverse approaches into a unified framework for comprehensive protection.

While HWTPM can effectively establish trust in embedded system networks, it requires a dedicated hardware chip for every node, which significantly increases deployment costs—especially since most commercial embedded systems lack built-in HWTPM support. In addition to the risk of physical compromise, the system must address intrusion threats from the open internet. To mitigate these risks, the proposed architecture monitors the behavior and integrity of each node, isolating any that show signs of modification or abnormal activity.

## 3. Background

This section introduces the core technologies that form the foundation of this research: Secure Boot, Measured Boot, Trusted Platform Module (TPM), and packet parsing.

### 3.1. Pseudo Linux Boot Process

**Figure 2:**
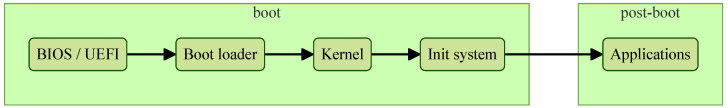
Boot sequence.

When a system is powered on, it first executes the Basic Input/Output System (BIOS) or the Unified Extensible Firmware Interface (UEFI). The boot loader is then launched, presenting the user with available boot media options. Once a boot medium is selected, it is loaded into system memory, and the operating system (OS) kernel is invoked. The kernel subsequently initiates the init process, which initializes the entire OS environment. The boot process is considered complete when the init system finishes its tasks and transfers control to the user [20], as illustrated in Figure 2.

Secure boot and measured boot are critical mechanisms for establishing trust in the system’s boot sequence by ensuring that each stage of the boot process remains unaltered and authentic. Although they serve different purposes, both can be implemented on the same machine to enhance overall system integrity. Secure boot establishes a chain of trust starting from the BIOS by allowing only signed and verified bootloaders and kernels to execute. In contrast, measured boot starts from the Trusted Platform Module (TPM), recording cryptographic measurements of each component loaded during boot. Together, these processes extend trust from the firmware or TPM to the entire system, helping to detect or prevent unauthorized modifications.

**Figure 3:**
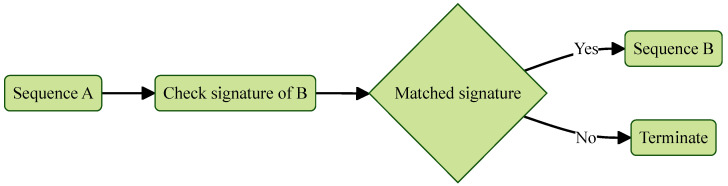
Secure boot partial sequence.

The root of trust for secure boot begins with the UEFI firmware, which is loaded from the onboard ROM. As illustrated in Figure 3, secure boot maintains trust through a sequence of verified execution transfers. At each stage of the boot process, the currently executing component verifies the digital signature of the next component before handing over control. If the signature is valid and the component remains unmodified, execution proceeds. Otherwise, the boot process is halted to prevent the execution of potentially compromised or unauthorized code. This mechanism ensures that only trusted and verified components are loaded throughout the boot sequence.

**Figure 4:**
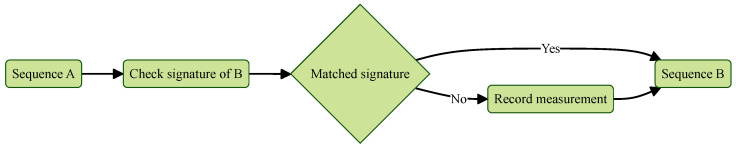
Measured boot partial sequence.

The key distinction between measured boot and secure boot lies in their response to potential tampering. As shown in Figure 4, measured boot does not interrupt the boot process—even if modifications are detected. Instead, at each stage of the boot sequence, the current component hashes the next component before execution and stores the hash in the Platform Configuration Registers (PCRs) of a Trusted Platform Module (TPM). If the hash matches a known good value, execution proceeds as expected. If it differs, the new hash is still recorded, replacing the previous one. This approach ensures that all changes—intentional or malicious—are measured and logged, but not blocked. Because measured boot only records these measurements, it requires a remote attestation mechanism with a trusted external platform to verify the stored hashes and detect any unauthorized changes or tampering.

### 3.2. Trusted Platform Module (TPM)

The Trusted Platform Module (TPM) exists in two major versions: TPM 1.2, released in 2016, and TPM 2.0, released in 2020. TPM 2.0 is the latest iteration, providing enhanced cryptographic capabilities such as support for Elliptic-Curve Cryptography (ECC) and adherence to improved security standards. Although TPM 2.0 shares many functionalities with TPM 1.2, it is not backward compatible with the earlier version [21].

TPM implementations come in various form factors. A discrete TPM (dTPM) is a hardware-based module embedded within an Application-Specific Integrated Circuit (ASIC), equipped with dedicated computational resources such as a CPU, memory, and cryptographic acceleration hardware. This hardware isolation enables the dTPM to securely store sensitive information independently of the host system, making it a trusted component even if the host platform is untrusted. In this work, dTPM is referred to as hardware TPM (HWTPM).

On the other hand, a virtual TPM (vTPM) is a purely software-based TPM implementation that adheres to the TPM specification. vTPMs are often deployed on trusted servers hosting Virtual Machines (VMs), where they serve as TPMs for the individual VMs. Since VMs are isolated from the host by hypervisors, the vTPM software is relatively protected from compromise originating from the hosted VMs. vTPMs are also valuable for TPM-related development due to their functional similarity to dTPMs [22]. In this study, vTPM is referred to as software TPM (SWTPM).

### 3.3. Network Traces and Packet Parsing

A unique network trace is identified by a stream of network traffic sharing the same source IP address, destination IP address, and protocol. To effectively detect and monitor ongoing traces, each network packet must be processed to extract key information—such as the source and destination IP addresses and the protocol type—allowing differentiation between multiple active traces within the network.

Even without analyzing the full content of each packet, monitoring the traces can still yield valuable insights. For instance, it can help identify abnormal network traffic patterns, which may indicate issues such as a malfunctioning device, receipt of unexpected traffic, or an unusually high volume of outgoing data. These anomalies can be detected by observing changes in the measurement matrix over time, such as fluctuations in the $F_{2}$ scores.

(1) $$F_{2}=\sum_{i=1}^{n=m}f_{i}^{2}=f_{1}^{2}+f_{2}^{2}+\ldots +f_{m}^{2}$$

The $F_{2}$ score of a data stream can be calculated using (1). When applied to a data stream with *m* distinct items, the $F_{2}$ score reflects the variation in the counts of these items over time.

Interpreting the distinct items as packet counts observed across different intervals, (1) captures changes in packet count distributions. A sudden deviation in this pattern within the observation window may indicate anomalous network traffic, signaling potential irregularities or attacks.

## 4. Proposed Methodology

This section presents an overview of the hardware, software, and the proposed architecture utilized in this research. It further provides a detailed explanation of both the server-side and client-side node designs. The complete software implementation of the proposed architecture is available at: https://github.com/CYCU-AIoT-System-Lab/TPM_Sharing_Scheme (accessed on 16 June 2025).

### 4.1. General Architecture

**Figure 5:**
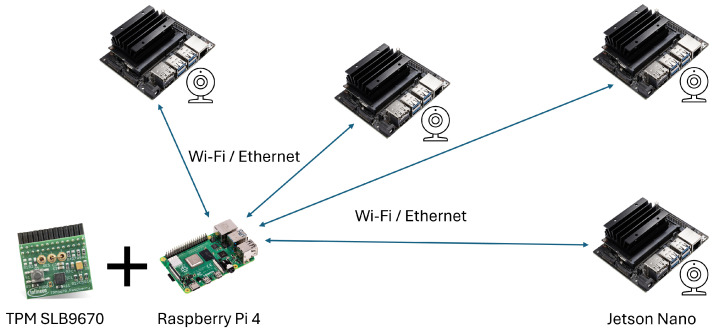
System hardware architecture representation of embedded system network deployed in this study.

The embedded system network used in this study comprises two widely adopted hardware platforms: the Raspberry Pi 4 Model B and the Jetson Nano, as illustrated in Figure 5. The Raspberry Pi is compatible with the Infineon (Neubiberg, Germany) OPTIGA^TM^ TPM SLB 9670VQ2.0 module and operates as the trusted platform within a secure environment. In contrast, the Jetson Nano, equipped with a GPU capable of handling AI and large-scale array processing workloads, functions as the untrusted platform deployed in an untrusted environment. Its integrity must therefore be verified and reported to the Raspberry Pi. All nodes in the embedded system network are interconnected via a single Wi-Fi router, forming a local area network (LAN).

**Figure 6:**
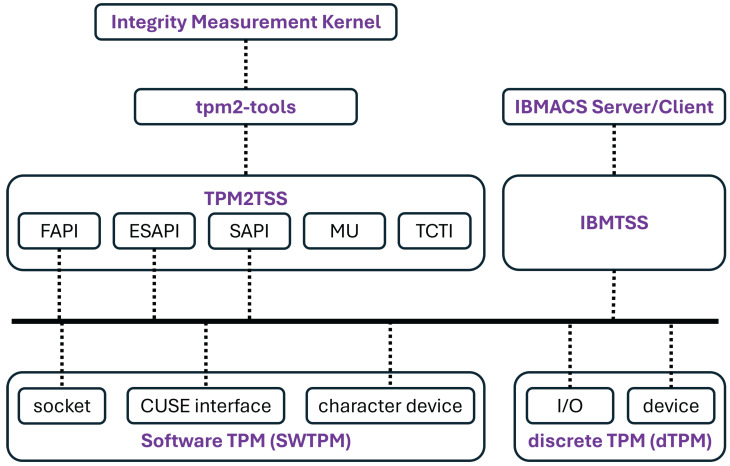
Common software stack relationship in both server and client nodes.

To fulfill the requirements of IBMACS, both the server and client nodes rely on the software components listed in Table 2. These components provide the essential dependencies for IBMACS and the Integrity Measurement Kernel, as depicted in Figure 6. It should be noted that only the major software components are presented; the complete list of dependencies is not included.

**Figure 7:**
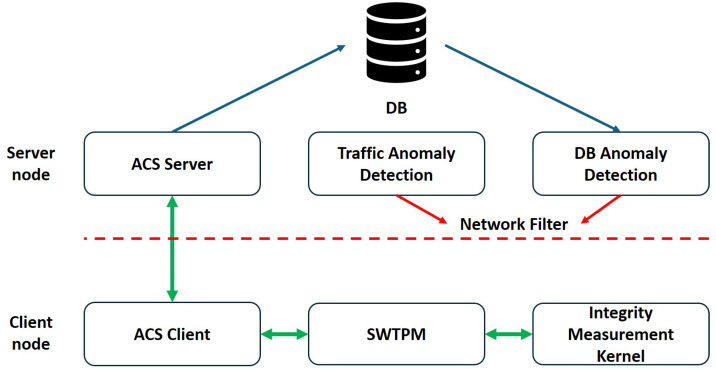
TPM sharing scheme architecture used in this study.

Since client nodes are assumed to operate in untrusted environments and cannot be considered reliable after deployment, they must continuously report their system integrity. As illustrated in Figure 7, IBMACS is deployed on the server node to handle client integrity reporting and record storage. After a client node completes its initial enrollment in a trusted environment, it can be deployed in an untrusted environment and consistently perform remote attestation (RA). A database (DB) anomaly detection (AD) agent on the server regularly inspects the RA records to identify any failures. Additionally, a traffic anomaly detection agent monitors traffic originating from each client node, flagging irregular patterns as potential signs of intrusion. If either agent detects an anomaly, a network filter is immediately deployed to block all traffic from the affected client node, thereby protecting the rest of the network.

**Figure 8:**
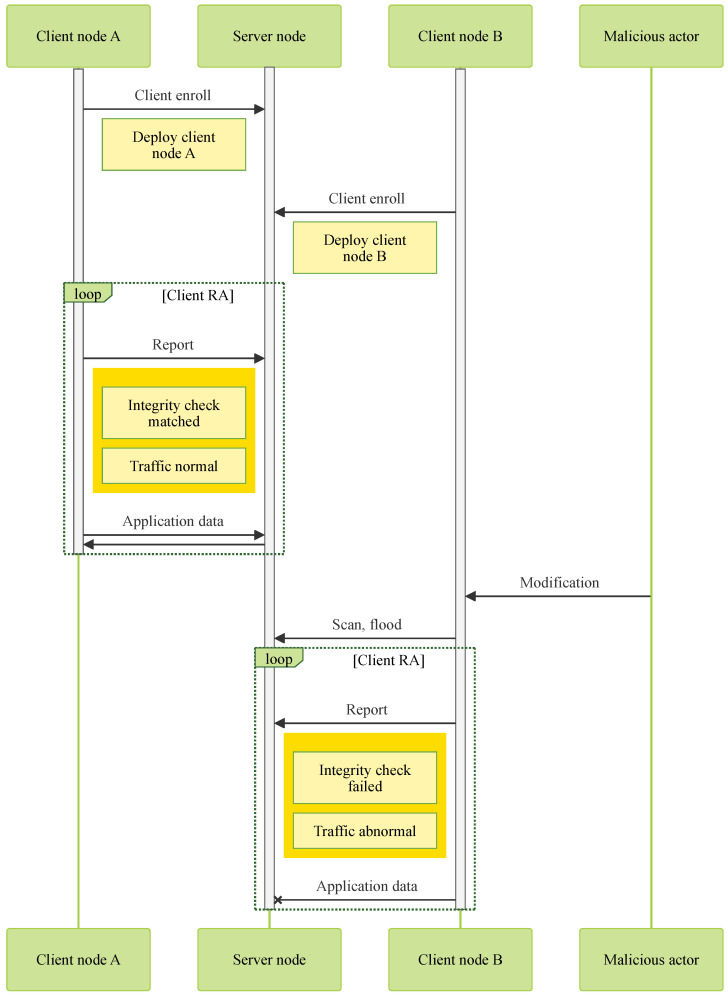
TPM sharing scheme architecture handling normal and malicious modified client nodes.

As shown in Figure 8, the system requires the server node to be operational with the IBMACS, DB anomaly detection (AD) agent, and traffic AD agent running. All client nodes—such as “Client node A” and “Client node B”—must complete an initial integrity report or client enrollment in a trusted environment before deployment. This process captures their unmodified state and ensures that all critical functionalities are working correctly.

Every time a client node tries to exchange information with the server node, an integrity report is required before doing so. If the integrity report is considered valid by the IBMACS, it will update DB with the new successful report record. The DB AD agent will read the latest report record and not take any action since no anomaly has been presented. In the meantime, the traffic AD agent has been monitoring individual client nodes throughout its uptime. A typical traffic profile needs to be set by the administrator for specific applications so that only traffic under a typical profile is allowed. In the case of an unmodified client node, its live traffic profile matches the set typical profile. Thus, no action is performed against the client node. In that instance, both agents do not find any anomaly; the client node can freely communicate with the server node to perform its original tasks like “Client node A” in Figure 8.

Each time a client node attempts to exchange information with the server node, it must first submit an integrity report. If the IBMACS verifies the report as valid, it updates the database (DB) with the new successful attestation record. The DB anomaly detection (AD) agent then reads this latest entry and takes no action, as no anomaly is detected. Concurrently, the traffic AD agent continuously monitors the traffic of individual client nodes. A typical traffic profile, predefined by the administrator for each application, serves as a baseline for acceptable behavior. In the case of an uncompromised client node, its live traffic aligns with the expected profile, and therefore, no action is taken. In such instances—illustrated by “Client node A” in Figure 8—both agents detect no anomalies, allowing the client node to communicate freely with the server node and carry out its intended tasks.

In the case of a compromised client node, modifications will be reflected in its regular integrity reports sent to the server prior to application communication. The IBMACS will fail to verify such reports, resulting in a failed attestation record being logged in the database (DB). Upon detecting this failed report, the DB anomaly detection (AD) agent will trigger network filtering to isolate all traffic originating from the compromised client node. Similarly, the traffic AD agent continuously monitors for irregular traffic patterns, such as network scanning, flooding, or other intrusion attempts, and will also isolate any suspicious traffic from the network. As illustrated in Figure 8, “Client node B” is effectively blocked from communicating with the server node following a failed integrity report or the detection of abnormal traffic.

**Figure 9:**
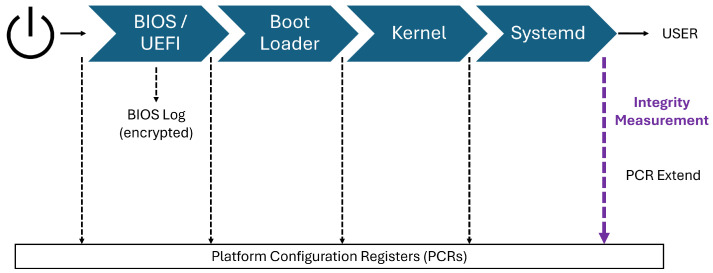
Integrity measurement kernel execution sequence on client nodes.

Client nodes running the IBMACS client use SWTPM instead of HWTPM. To enhance boot-time security, a custom measurement kernel is activated to measure any mission-critical software prior to execution, as illustrated in Figure 9. This kernel records the integrity measurement digest in the SWTPM’s PCR bank, which the IBMACS client then retrieves and reports to the server node for verification.

### 4.2. Server Node

The server node is built upon IBMACS to establish a robust integrity reporting system. It acts as the central information exchange hub for the entire embedded system network and serves as the gatekeeper of network security. Using IBMACS, the server continuously collects integrity data from client nodes while also monitoring their network traffic. If a client node’s integrity is compromised or it generates abnormal traffic, the server promptly isolates that node to prevent further impact on the network.

**Figure 10:**
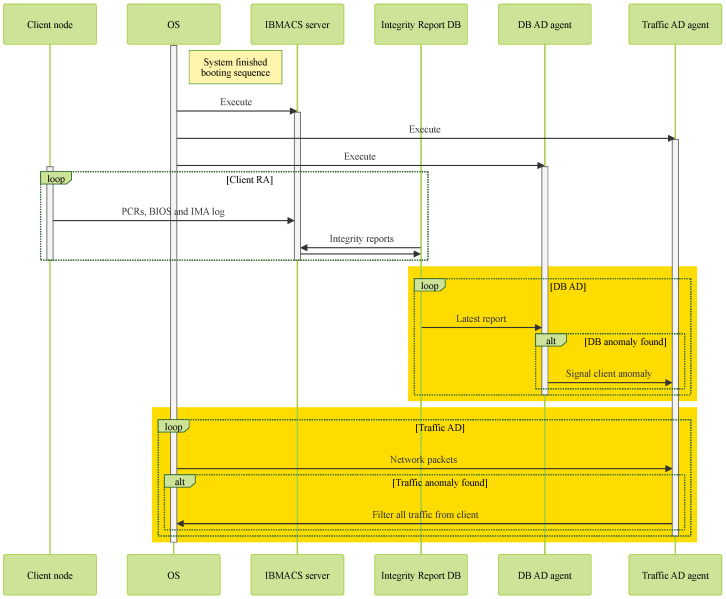
Server architecture.

As shown in Figure 10, the IBMACS, DB AD agent, and traffic AD agent are launched once the OS completes booting. The IBMACS is responsible for receiving and validating integrity measurement reports from client nodes, which include PCR values from their PCR bank, BIOS logs, and IMA logs. Regardless of whether the report is valid or indicates a modification in the client, the validation result is recorded as the latest entry in the database.

The DB AD agent parses the latest database entry and, if the entry does not correspond to a valid client node report, it sends a Linux user-defined signal to the traffic AD agent to trigger the deployment of a filter against the abnormal client.

Once launched, the traffic AD agent continuously monitors traffic from the specified client node. If the observed traffic profile deviates from the typical profile defined by the network administrator, the agent will deploy a network filter to block all traffic from the monitored client node. Additionally, upon receiving the Linux user-defined signal—indicating a failed integrity check and a potentially compromised client—the traffic AD agent will immediately deploy the network filter.

#### 4.2.1. DB Anomaly Detection (AD)

The DB AD agent queries the IBMACS database, which contains data structured as shown in Table 3, and retrieves the most recent entry in the format presented in Table 4. This approach enables the DB AD agent to obtain the latest integrity measurement report status and verify its validity. If the status is deemed invalid, the DB AD agent sends a Linux user-defined signal to the traffic AD agent, prompting it to isolate the corresponding client node.

#### 4.2.2. Traffic Anomaly Detection (AD)

**Figure 11:**
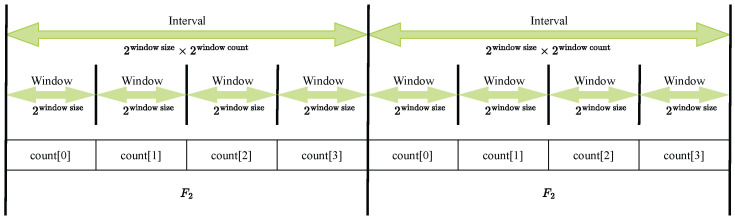
Traffic AD intervals, windows, and $F_{2}$ scores.

In Figure 11, an interval consists of multiple windows. The packets arriving within the timespan of each window are counted and assigned to corresponding buckets labeled count[0] through count[3]. At the end of each interval, these packet counts are used to calculate the $F_{2}$ score.

#### 4.2.3. Network Traffic Filter

In certain scenarios, an infiltrated node within embedded system networks may require immediate termination. This study employs network filters as a method to disable compromised nodes. Since the traffic AD agent is responsible for deploying system-wide filters against the monitored client node, this research utilizes Linux kernel netfilters to drop all packets originating from the affected client nodes.

### 4.3. Client Node

The client nodes are built on top of IBMACS, enabling the IBMACS client to send integrity measurement results to the server node. Previously installed SWTPM on client nodes only interfaces with IBMTSS, but to utilize the tpm2-tools utilities needed for a custom measurement kernel, SWTPM must also interface with TPMTSS. Therefore, an updated version of SWTPM is required on client nodes. With the measurement kernel in place, it can measure the applications deployed on client nodes and update the measurement digest in SWTPM’s PCR registers. Subsequently, the IBMACS client sends the integrity measurement report to the server node.

**Figure 12:**
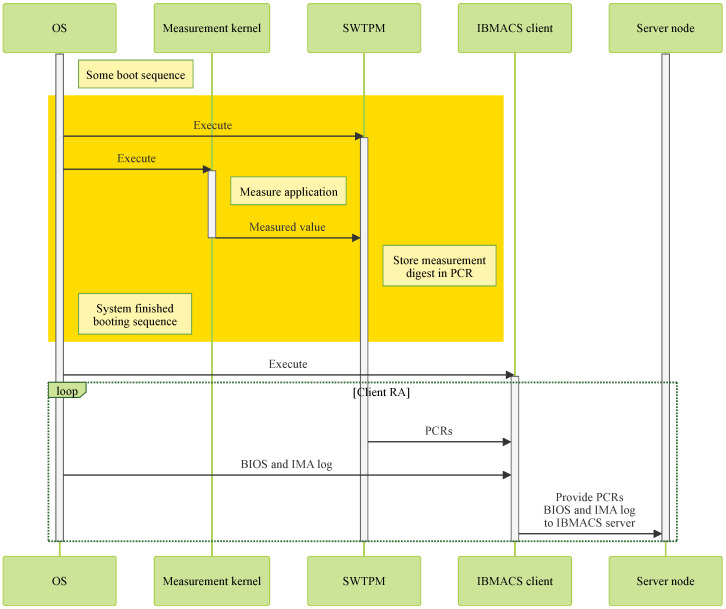
Client architecture.

As shown in Figure 12, once the OS completes the boot sequence, the init system launches both SWTPM and the measurement kernel. SWTPM handles requests from IBMTSS and TPMTSS, while the measurement kernel measures files and applications prior to execution. When necessary, it uses tpm2-tools utilities to update the measurement digest accordingly.

Before any deployed application on a client node communicates with the server node, a successful remote attestation (RA) must be completed. The IBMACS client collects PCR values from SWTPM, along with BIOS and IMA logs from the OS, and sends these data to the server node for verification.

#### 4.3.1. Up-to-Date SWTPM

The operating system used on the Jetson Nano in this study is based on Ubuntu 18.04, released in 2019. Many required dependencies are either unavailable in the system’s APT repository or are outdated, necessitating the compilation of numerous software packages from source. Some of these dependencies are also required by core Linux utilities. Installing them system-wide risks breaking critical applications, including wget and IBMACS. Therefore, minimizing and avoiding system-wide installation of dependencies is essential. Table 5 lists the dependencies needed to install an up-to-date version of SWTPM on the Jetson Nano, while additional software in Table 6 can be installed to replace older counterparts.

#### 4.3.2. Measurement Kernel

**Figure 13:**
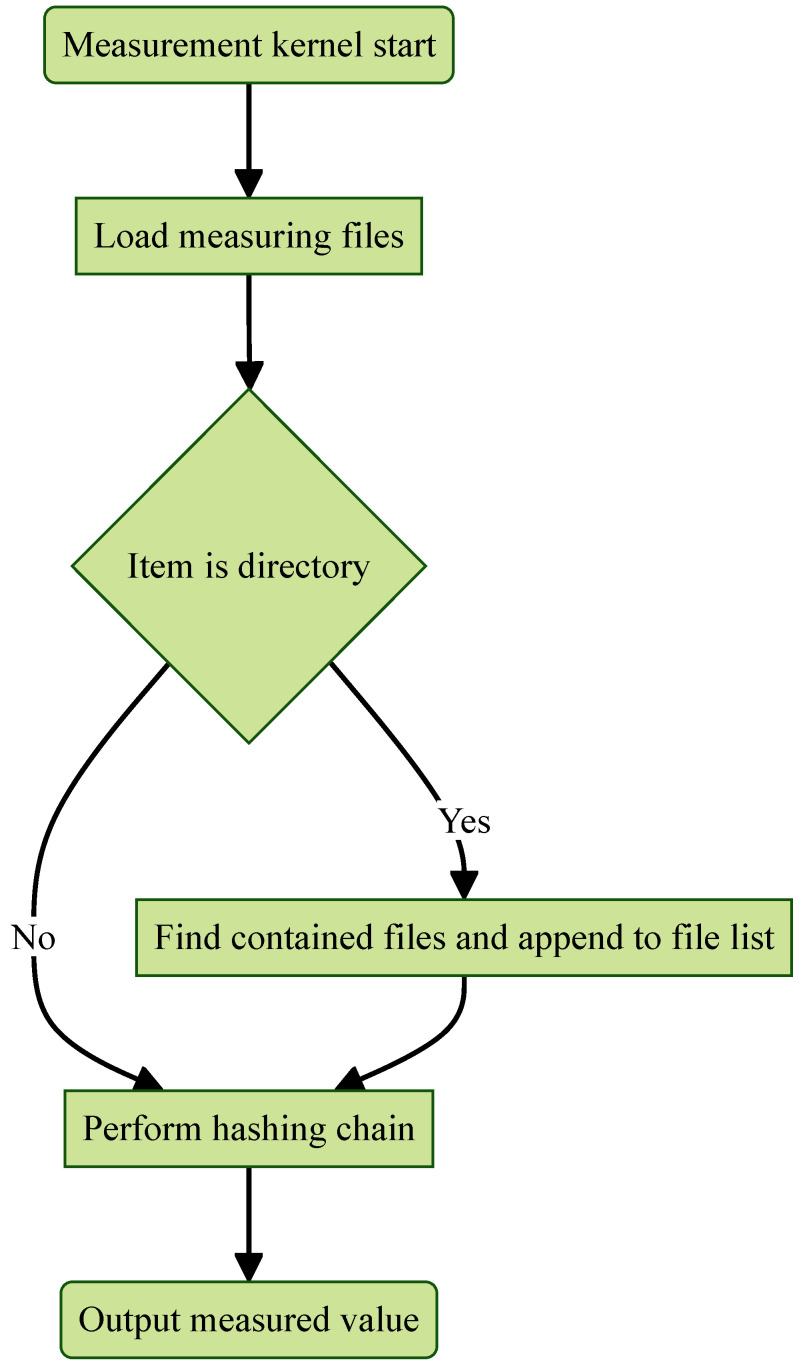
Measurement kernel flowchart.

When executed, the measurement kernel loads files and directories to be measured from a predefined file. It then recursively discovers all files within the specified directories and appends them to the existing list of files, as illustrated in Figure 13. The measurement kernel begins by hashing the first file using ${\mathrm{PCR}}_{x-1}$, the last PCR value utilized by the OS during the boot sequence. This hashing chain ensures that the final measured value depends on the boot sequence; any change in the previous PCR will alter the measured value, as described in (2). After computing the initial measurement $h_{1}$, the kernel sequentially measures each file in the list, combining it with the previous measurement $h_{n}$ to derive the final measurement value $h_{x}$.

(2) $$\begin{array}{cc} h_{1} & =\mathrm{SHA}\text{-}256({\mathrm{PCR}}_{x-1}||\mathrm{File}\;1)\\ h_{2} & =\mathrm{SHA}\text{-}256(h_{1}||\mathrm{File}\;2)\\ h_{3} & =\mathrm{SHA}\text{-}256(h_{2}||\mathrm{File}\;3)\\ \; & \vdots \\ h_{n} & =\mathrm{SHA}\text{-}256(h_{n-1}||\mathrm{File}\;n) \end{array}$$

The measurement kernel is designed to detect any modifications to the files specified by the system administrator. Thanks to the properties of cryptographic hash functions, even a small change in these files will produce a different measured value. For a malicious actor to generate the same measured value—a hash collision—they would need to find a combination of file manipulations that simultaneously executes malicious code while producing an identical hash. Such an attack is computationally infeasible due to the strong collision resistance of the hash function.

#### 4.3.3. Measured Boot Chain (MBC) Appending

**Figure 14:**
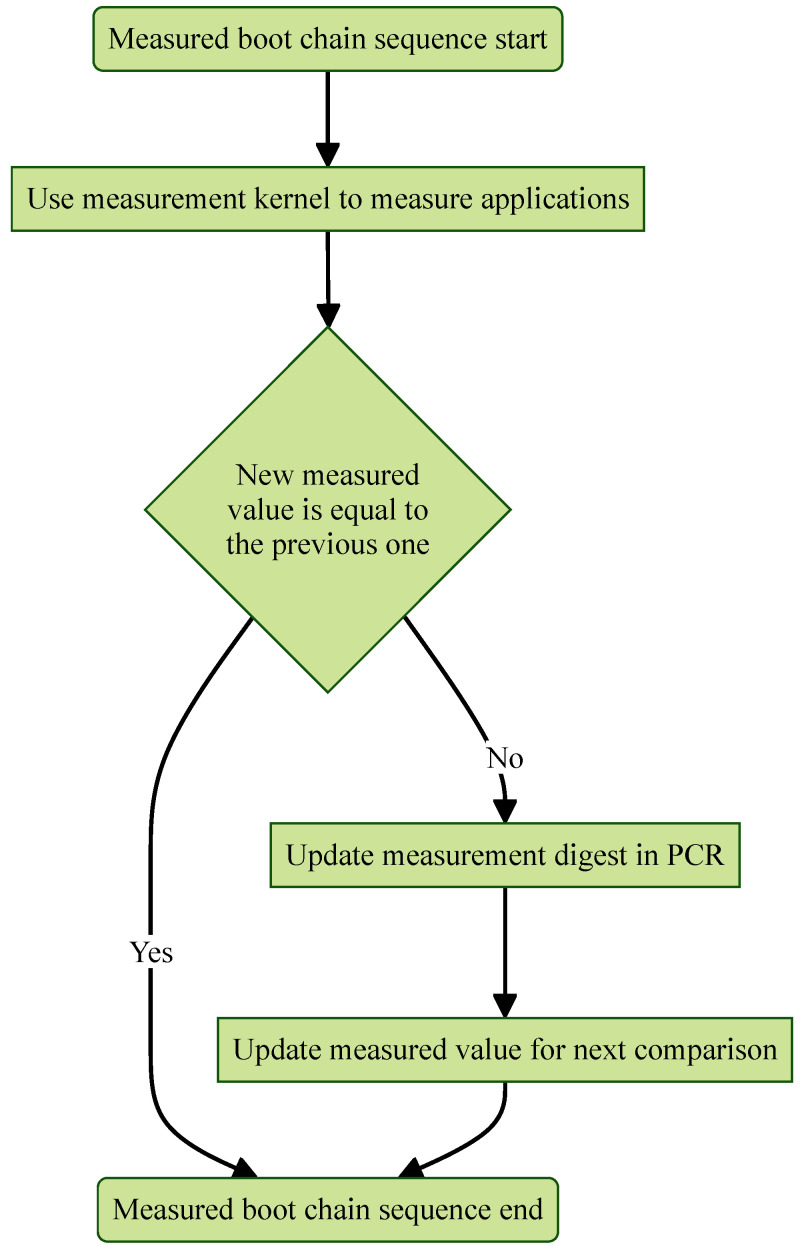
Activate measurement kernel with MBC and update PCR.

The custom measurement kernel is launched at the end of the boot sequence, completing the Measured Boot Chain (MBC). If the newly computed measured value matches the one from the previous boot, the MBC concludes as shown in Figure 14. However, if the two values differ, the new measurement is used to update the measurement digest according to (3), replacing the prior value.

(3) $$\begin{array}{cc} {\mathrm{PCR}}_{x} & =\mathrm{PCRExtend}(\mathrm{Measured}\;\mathrm{value}) \end{array}$$

A malicious actor might attempt to alter the previously generated measured value stored locally to prevent the MBC activation agent from updating the measurement digest. To counter this, a copy of the newly computed measured value should be sent to the server node, enabling it to detect any modifications even if the local measurement digest remains unchanged.

## 5. Experiments and Evaluation

### 5.1. IBMACS Consistent Remote Attestation (RA)

**Figure 15:**
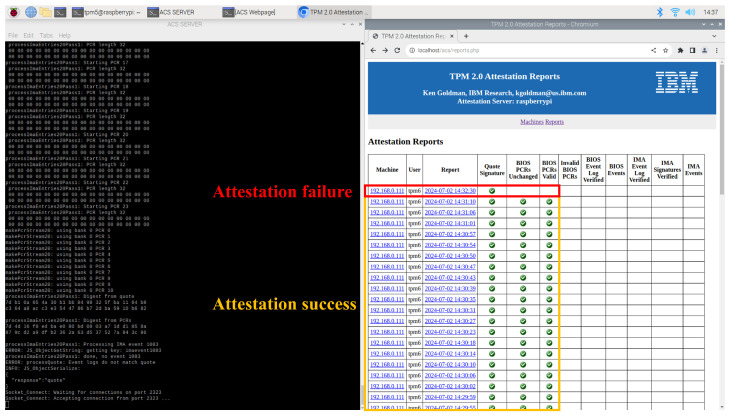
Consistent RA from the same client node.

In the experiment evaluating whether IBMACS can perform consistent RA, the client node was able to attest its integrity reliably at regular intervals—even prior to each communication initiated by the deployed application. As shown in the web UI on the right in Figure 15, the client node successfully completed attestation from “14:29:55” to “14:31:10”. The machine with IP address “192.168.0.111” repeatedly performed RA at 3 to 4 s intervals, all yielding successful results. In contrast, the entry recorded at “14:32:30” demonstrates an example of a failed attestation attempt.

### 5.2. IBMACS Database (DB) Anomaly Detection (AD)

**Figure 16:**
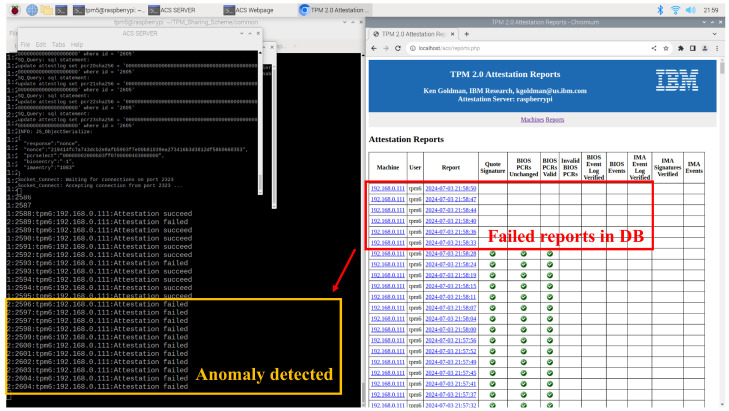
DB AD agent detects anomaly in DB and exports different status.

The DB AD agent on the server node parses the latest report entry to detect anomalies and determines the status based on the rules defined in Table 7. The status of “1” indicates a client enrollment event, requiring no further action. The status of “2” signifies a successful attestation, also requiring no action. The status of “3” denotes any other condition, such as an unexpected event or a failed attestation attempt. As shown in Figure 16, and in accordance with Algorithm 1, the client node is identified as anomalous after the timestamp “21:58:28”. When the DB AD agent outputs status “3”, a Linux user-defined signal is sent to the traffic AD agent, which then deploys a network filter to isolate the client node.
**Algorithm 1** Trigger traffic AD to deploy filter**if** Status=1 **then**   Normal **else if** Status=2 **then**   Normal **else if** Status=3 **then**   Abnormal,sendSIGUSR1 **end if**

### 5.3. Traffic Anomaly Detection (AD)

The traffic AD agent is designed to detect abnormal traffic by analyzing changes in the $F_{2}$ value between monitoring intervals. If abnormal behavior is identified, a network filter is deployed to isolate the monitoring client node. Additionally, the agent will deploy a filter upon receiving a Linux user-defined signal, SIGUSR1, from the DB AD agent

#### 5.3.1. Parameter Selection and Approach for Faster Response

**Figure 17:**
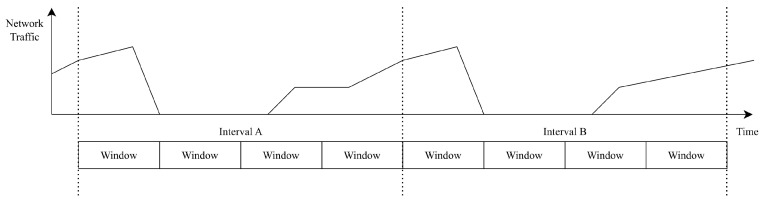
Traffic AD agent and parameter selection.

When configuring the parameters—window size, window count, and $F_{2}$ deviation threshold—the administrator should first analyze the typical traffic patterns between the client and server nodes, as illustrated in Figure 17. A recommended practice is to ensure that repeating traffic patterns are contained within a single window. If a pattern spans a longer duration, it is acceptable to fit it within a single interval instead. The $F_{2}$ deviation threshold should be set based on the maximum observed deviation during testing, with additional headroom to account for fluctuations caused by network latency.

**Figure 18:**
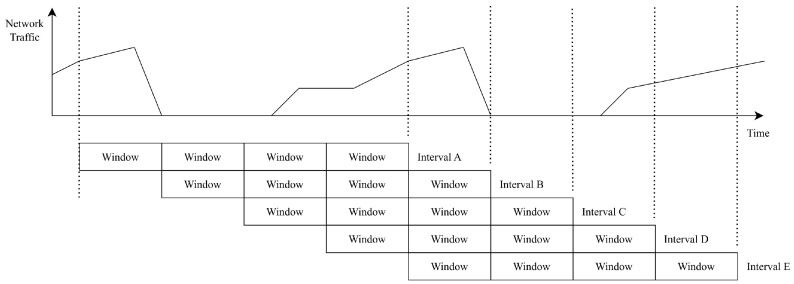
Optimize traffic AD agent for faster response.

In the current implementation of the traffic AD agents, anomalies are only reported at the end of each monitoring interval. Depending on the configured settings, this may introduce a significant time window during which malicious activities can occur undetected. To mitigate this, the detection process can be optimized by overlapping intervals and recalculating deviations each time a new window of data is collected, as illustrated in Figure 18.

#### 5.3.2. Abnormal Traffic

The traffic AD agent calculates the $F_{2}$ value using (1) and determines the deviation between measurement windows using (4). As shown in Algorithm 2, an anomaly is detected—and a network filter is deployed—only if the computed $F_{2}$ deviation exceeds the predefined threshold.

(4) $$\begin{array}{cc} F_{2}\;\mathrm{deviation} & =|F_{2}\left(n\right)-F_{2}\left(n-1\right)| \end{array}$$

**Algorithm 2** Filter deployment**if** $F_{2}\;\mathrm{deviation}>\;\mathrm{threshold}$ **then**   Abnormal,deployfilter **else**   Normal **end if**

**Figure 19:**
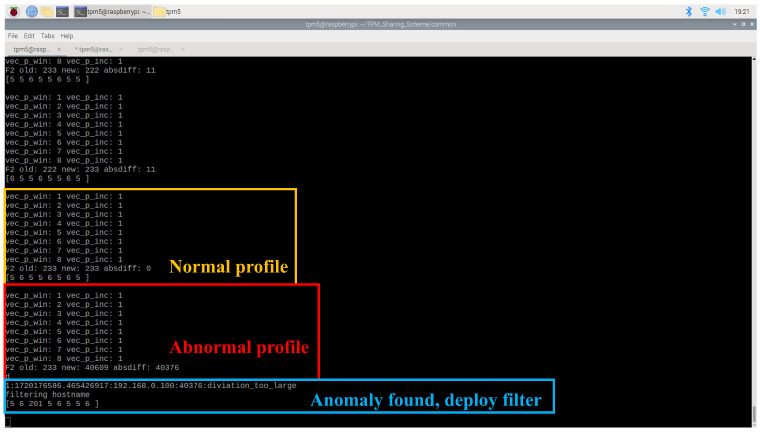
Traffic AD agent found traffic profile is abnormal and deployed network filter.

An experiment was conducted to validate the anomaly detection capabilities of the traffic AD agent. In the normal scenario, a client node continuously pinged the server node at 0.2 s intervals to generate a baseline traffic profile, as shown in Figure 19. The traffic AD agent was configured with a window size of $2^{30}=1073741824\approx 1\times 10^{9}$ nanoseconds, a window count of $2^{3}=8$, and an $F_{2}$ deviation threshold of 1000. Under normal conditions, the computed $F_{2}$ score remained around 200. However, when the client node initiated a ping flood, the $F_{2}$ score spiked to 40,609 (5), exceeding the defined threshold. As a result, the agent deployed a network filter to isolate the anomalous client node.

(5) |40609−233|=40378>1000

#### 5.3.3. Receive System Signal

**Figure 20:**
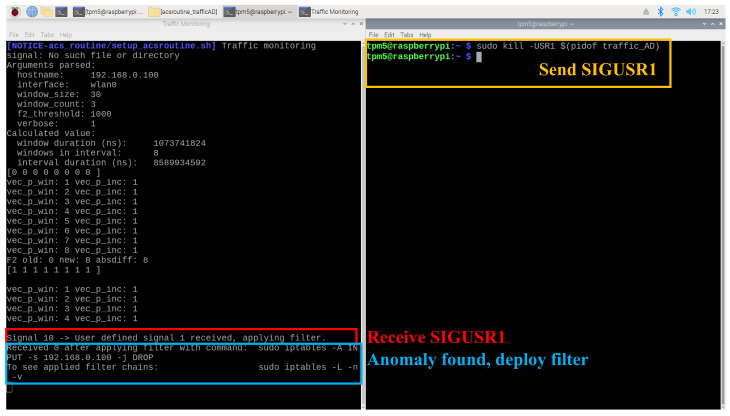
Traffic AD agent received Linux user-defined signal and triggers deployment of network filter.

The traffic AD agent is also designed to handle the SIGUSR1 signal as a trigger to deploy a network filter. Once the agent is launched, receiving a SIGUSR1 signal prompts it to deploy a filter against the monitoring client node. As shown in Figure 20, the agent correctly responds to the signal. In the right terminal, SIGUSR1 is sent using the Linux kill utility after the traffic AD agent has started. In the left terminal, the agent successfully handles the signal and deploys a network filter to isolate the monitoring client.

#### 5.3.4. Deploy Filter

**Figure 21:**
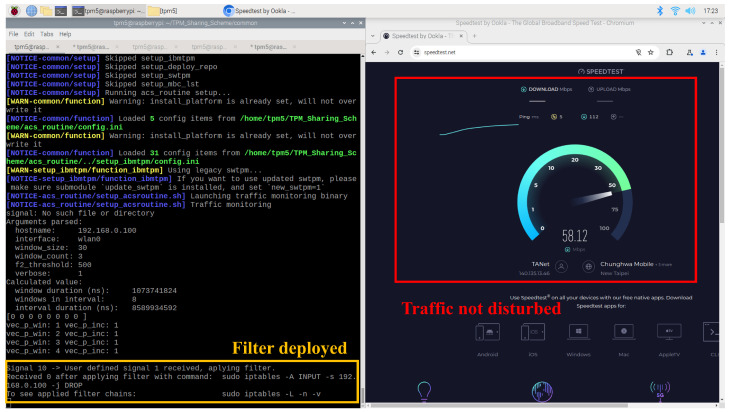
Deployed filter only affects monitoring client node but not the entire network traffic.

The network filter deployed to isolate the monitoring client node does not impact the server node’s network connectivity. As illustrated in Figure 21, the filter does not interfere with the Raspberry Pi’s connection to a network speed testing website. Instead, it selectively blocks communication with other client nodes as a means of disabling compromised devices.

### 5.4. Measurement Kernel

**Figure 22:**
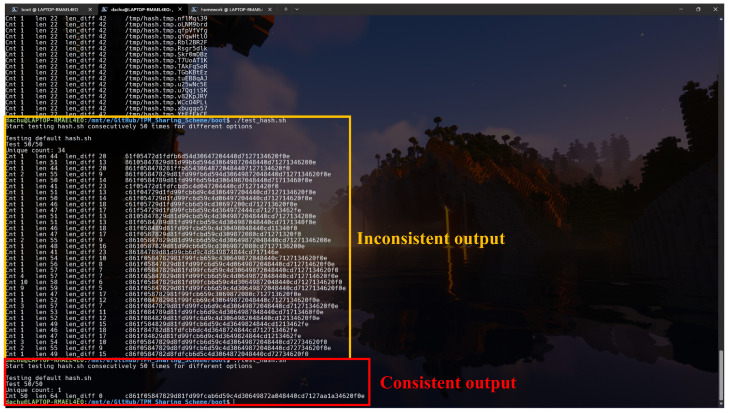
Consistency of measured value computed by measurement kernel.

The primary task of the measurement kernel is to produce distinct measurement values when input files are modified while maintaining consistent output for unaltered files. Therefore, verifying the consistency of the measurement kernel is critical. As shown in Figure 22, output inconsistencies are highlighted in the center of the figure. After refining the kernel implementation, the measurements remain consistent throughout the test when applied to unchanged files.

**Figure 23:**
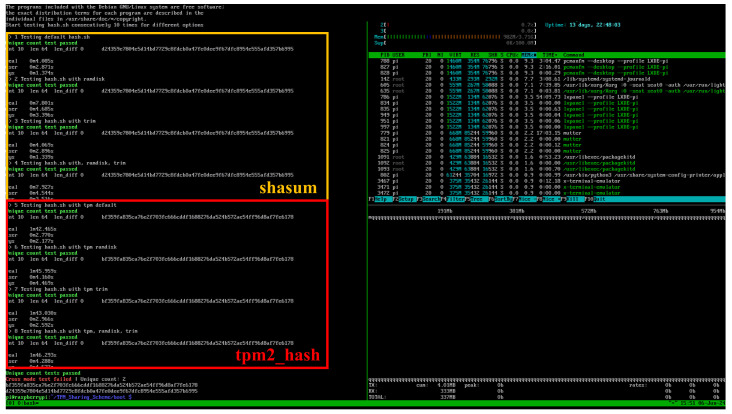
Result comparison between different binaries used.

By default, the measurement kernel utilizes the shasum hashing utility provided by standard Linux tools. Alternatively, it can employ the tpm2_hash utility from tpm2-tools, which leverages the TPM’s internal hashing engine. As illustrated in Figure 23, the top four tests use shasum and the bottom four use tpm2_hash; both methods yield consistent outputs. Nevertheless, shasum is preferred to avoid consuming TPM computational resources during routine measurements.

**Figure 24:**
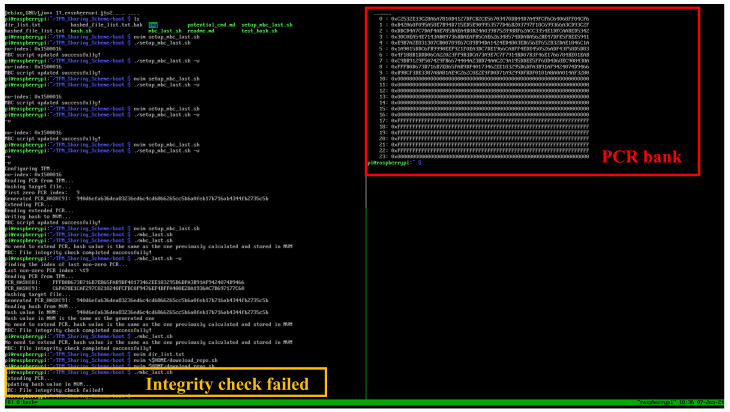
Demonstration of an MBC integrity check failure output.

If the measured value generated by the measurement kernel differs from the previously recorded value, an agent will update the measurement digest stored in the Platform Configuration Registers (PCRs) using the new value. As shown in Figure 24, the bottom-left corner illustrates the agent invoking the measurement kernel, detecting file modifications, and subsequently updating the measurement digest. On the right, the updated contents of the PCR bank are displayed following this operation.

**Figure 25:**
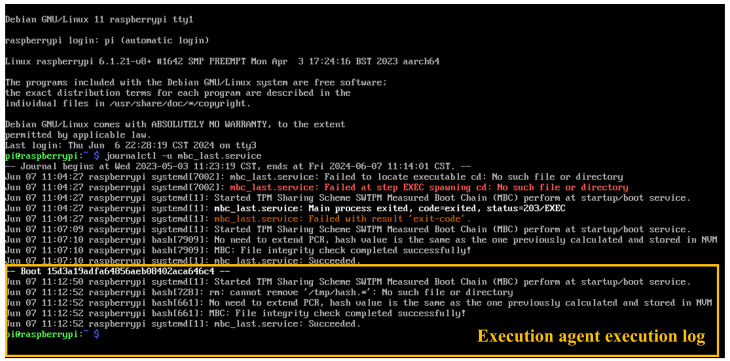
Measurement kernel activated after system boots with integrity check completed.

On the other hand, the measurement kernel must be launched during the boot process to complete the MBC. As shown in Figure 25, the message generated by the measurement kernel and its execution agent confirms successful execution following the boot event identified by the hash 15d3a19adfa64856aeb08402aca646c4.

## 6. Conclusions

This study focuses on creating an optimum solution to use the proposed TPM sharing scheme architecture to protect the integrity of embedded system nodes. The server node is equipped with HWTPM and IBMACS to verify and keep a record of the integrity of the client nodes. DB and traffic AD agents are responsible for isolating breached client nodes. Each client node has a custom measurement kernel to monitor deployed applications. If these applications are modified, the client node would then be identified by the server node and isolated from the rest of the network. In the experiments, the IBMACS can monitor client nodes and frequently ensure system integrity before the deployed application interacts with the rest of the system. The custom measurement kernel can perform consistent measurement and is activated on boot by an agent to update measurement digest if needed. The BD AD agent can parse the latest entry to find failed attestation attempts. The traffic AD agent filters out all traffic from the monitoring client node if the traffic profile is abnormal or signaled by the BD AD agent with a failed attestation attempt. While the proposed agent is capable of detecting traffic surges, its effectiveness can be further enhanced by incorporating more recent detection techniques. In its current form, the agent can drop all traffic originating from nodes flagged as anomalous, thereby reducing the computational overhead typically incurred during DDoS attacks. Overall, the proposed architecture achieved the following:

1. Adopted SWTPM on the client nodes to lower deployment costs.
2. Adopted IBMACS for the client nodes’ integrity reporting.
3. Implemented a DB AD agent to observe anomalies and signal traffic AD agent to isolate the monitoring client node on the server node.
4. Implemented a traffic AD agent to find abnormal traffic, handle the user-defined signal, and deploy filters to isolate the monitoring client node on the server node.
5. Implemented an integrity measurement kernel with consistent results to measure applications deployed on the client nodes.
6. Implemented an integrity measurement kernel activation agent that executes on boot and updates the measurement digest to extend MBC on the client nodes.

Several improvements can be made to further enhance the proposed client architecture. Implementing and extending the coverage of secure boot can ensure that the SWTPM remains unmodified, ensuring the system can only operate with a trusted SWTPM [15]. Accelerating the measurement kernel through parallel processing can reduce the time required for a client node to be ready for Remote Attestation (RA). Additionally, monitoring both the SWTPM and deployed applications with Integrity Measurement Architecture (IMA) can provide a more comprehensive view of modifications, reflected not only in Platform Configuration Registers (PCRs) but also in the IMA event log.

For the server-side architecture, adopting a replica database can prevent incomplete entries from being read by the database anomaly detection (DB AD) agent, avoiding potential misjudgments. Furthermore, incorporating algorithms such as Bloom filters [23], Count-Min sketch [24,25,26], hCount [24,27], or timestamp-vector [28] can enable efficient monitoring of a large number of IP addresses. This approach can reduce the need to execute traffic AD agents for all client nodes in the network. While this study presents a lightweight traffic AD framework for embedded systems, further work can be conducted to benchmark and improve the proposed approach against existing state-of-the-art methods. In particular, future efforts should include a comparative analysis using common datasets and metrics that reflect detection accuracy, false positive rates, latency, and computational overhead specifically in the context of embedded system networks.

The traffic AD agent monitors traffic patterns between client nodes and the system backend, effectively detecting sudden surges or deviations indicative of possible distributed attacks. However, this mechanism may produce false positives in legitimate scenarios such as large-scale client updates or synchronized backup operations, which can temporarily resemble attack behavior. Similarly, the measurement kernel monitors system configurations and critical files to detect tampering or unauthorized changes. While this enhances the system’s ability to detect compromise, it may incorrectly flag benign changes—such as temporary log file updates or legitimate configuration adjustments—as intrusions. These false positives highlight a trade-off between sensitivity and operational tolerance. Future work could also include integrating contextual awareness or whitelisting strategies to reduce erroneous alerts while maintaining strong coverage.

## Figures and Tables

**Table 1:** Approaches comparison. ✔: supports, ✘: does not support, ✽: unable to verify, ▲: functionally similar.

Item	Cao et al. [18]	Wang and Yan [15]	Lu et al. [16]	This study
OS protection	✘	✔	✔	✔
Custom applications protection	✘	✘	✽	✔
Data protection	✔	✘	✽	✔
SWTPM	✘	✘	▲	✔
Integrity reporting	✘	▲	▲	✔
Identify network anomaly	✘	✘	✘	✔

**Table 2:** Mutual software requirements for both the server and client nodes.

Software	Version
Raspberry Pi OS	2023-05-03-raspios-bullseye-arm64
Jetpack	Jetson-nano-jp461
tpm2-tss	3.2.0
tpm2-tools	5.2
tpm2-abrmd	2.4.1
tpm2-tss-engine	1.1.0
Optiga-tpm-explorer	1.1.4
IBMTSS	1.6.0
SWTPM	1682
IBMACS	1658

**Table 3:** DB example table. The dots are used to indicate that the pattern continues.

Id	Colume-1	Colume-2	…	Colume-n
1	$r_{1}c_{1}$	$r_{1}c_{2}$	…	$r_{1}c_{n}$
2	$r_{2}c_{1}$	$r_{2}c_{2}$	…	$r_{2}c_{n}$
⋮	⋮	⋮	⋱	⋮
*m*	$r_{m}c_{1}$	$r_{m}c_{2}$	…	$r_{m}c_{n}$

**Table 4:** Query result of DB example table.

Id	Colume-1	Colume-n
*m*	$r_{m}c_{1}$	$r_{m}c_{n}$

**Table 5:** Up-to-date SWTPM dependencies.

Software	Version	Software	Version
autoconf	2.72	libtool	2.4.7
automake	1.16.3	libtpms	0.9.6
bison	3.8.2	libunistring	1.2
expect	5.45.4	m4	1.4.19
flex	2.6.3	meson (pip package)	1.4.1
gawk	5.3.0	nettle	3.10
gettext	0.22.5	ninja (pip package)	1.11.1
glib	2.80.3	openssl	1.1.1w
gmp	6.3	p11-kit	0.25.3
gnutls	latest	packaging (pip package)	24.1
gperf	3.1	pkg-config	0.29.2
help2man	1.49.3	python3	3.12.4
json-c	0.17	setuptools (pip package)	70.1.1
json-glib	1.8.0	socat	1.8.0.0
libcurl	8.8.0	swtpm	0.9.0
libev	4.33	tcl	8.6.14
libffi	3.4.6	tcsd	0.3.15
libpcre2	10.44	texinfo	7.1
libseccomp	2.5.5	tpm2-tss	4.1.3
libtasn1	4.19.0	util-linux (libuuid)	2.40.1

**Table 6:** Up-to-date SWTPM-related software.

Software	Version
tpm2-abrmd	3.0.0
tpm2-tools	5.7
tpm2-tss-engine	1.2.0

**Table 7:** DB AD agent output status versus attestation reports stored in DB.

Agent Output		Attestation Reports		
**Status**	**Description**	**Quote Signature**	**BIOS PCRs Unchanged**	**BIOS PCRs Valid**
		**Quoteverified**	**Pcrschanged**	**Pcrinvalid**
1	Client enroll	1	0	NULL
2	Attestation success	1	0	0
3	Attestation failure	Else		

## Data Availability

The original contributions presented in this study are included in the article. Further inquiries can be directed to the corresponding author(s).

## Abbreviations

The following abbreviations are used in this manuscript:

AD	Anomaly Detection
BIOS	Basic Input/Output System
CSP	Cloud Service Provider
DAA	Direct Anonymous Attestation
DB	database
DDH	Decisional Diffie–Hellman
DDoS	Distributed Denial of Service
E3s	Encrypted Execution Environments
ECC	Elliptic-Curve Cryptography
HCE	Host Card Emulation
HWTPM	Hardware Trusted Platform Module
IBMACS	IBM Attestation Client Server
IDSs	Intrusion Detection Systems
LAN	Local Area Network
MBC	Measured Boot Chain
NAP	Node Authentication Protocol
NTESDs	Non-TPM deployed Embedded Smart Devices
PCR	Platform Configuration Register
RVP	Remote Verification Protocol
SEs	Secure Elements
SWTPM	Software Trusted Platform Module
SoC	System-on-Chip
TBP	Trusted Booting Protocol
TCG	Trusted Computing Group
TEEs	Trusted Execution Environments
TPM	Trusted Platform Module
TXT	Intel Trusted Execution Technology
UEFI	Unified Extensible Firmware Interface
VM	Virtual Machine
dTPM	discrete Trusted Platform Module
vTPM	virtual Trusted Platform Module
